# Neuroticism Drives Associations Between Repetitive Behaviors and Depression in Autistic Adults

**DOI:** 10.3389/fpsyt.2022.803361

**Published:** 2022-03-02

**Authors:** Jessica M. Schwartzman, Zachary J. Williams, Jared K. Richards, Samantha R. Mattheiss, Katherine O. Gotham

**Affiliations:** ^1^Department of Psychiatry and Behavioral Science, Vanderbilt University Medical Center, Nashville, TN, United States; ^2^Medical Scientist Training Program, Vanderbilt University School of Medicine, Nashville, TN, United States; ^3^Department of Hearing and Speech Sciences, Vanderbilt University Medical Center, Nashville, TN, United States; ^4^Vanderbilt Brain Institute, Vanderbilt University School of Medicine, Nashville, TN, United States; ^5^Frist Center for Autism and Innovation, Vanderbilt University, Nashville, TN, United States; ^6^Department of Psychology, Rowan University, Glassboro, NJ, United States

**Keywords:** autism spectrum disorder, depression, insistence on sameness, neuroticism, repetitive sensorimotor

## Abstract

Depression is more prevalent among autistic adults than neurotypical adults, yet risk factors are incompletely understood in this population. Some research groups have focused on relationships between negative repetitive thinking and depression in the autistic population, which may explain elevated prevalence rates in line with general population findings on rumination and internalizing disorders. Little is known about associations between depression and more prototypical repetitive cognitions and/or behaviors characteristic of autism (i.e., insistence on sameness [IS] and repetitive sensorimotor [RSM] behaviors). Therefore, the present study aimed to examine associations between IS, RSM behaviors, and depressive symptoms in 762 autistic adults, and whether observed effects are confounded by additional factors (e.g., demographic factors, trait neuroticism). To test if greater IS scores were associated with greater depressive symptoms on the BDI-II, a Bayesian linear regression was conducted with BDI-II scores (dependent variable) regressed on age, gender, educational level, RSM scores, and IS scores (independent variables). To test the effects of neuroticism on observed relationships, a second regression was conducted that included all predictors from the baseline model and neuroticism. Standardized regression coefficients were tested against an interval null hypothesis of [−0.1, 0.1] to assess for practical significance. Results indicated that IS exhibited a moderate positive relationship with depressive symptoms, while RSM behaviors provided only a slight increase in predictive ability. However in the second model, neuroticism exhibited a strong positive relationship with depressive symptoms, completely attenuating the effect of IS. Associations between RSM behaviors and depressive symptoms did not meet our criteria for practical significance, particularly when neuroticism was added to the model. Neither RSM nor IS moderated the effect of neuroticism on depression. The findings from this study add to the literature on risk factors in the pathway to depression in autism, and suggest opportunities for clinical translation to screening and intervention efforts. Screening for IS in autistic individuals is a common diagnostic practice in clinical and research settings that may be leveraged to also identify those at higher risk for depression, and increasing flexibility in daily life may promote emotional regulation and distress tolerance.

## Introduction

Compared to the general population, autistic adults face substantial risks for current and lifetime depression and suicidality (1–3). Given that depression is a leading public health concern worldwide (4) and is linked to reduced quality of life (5), efforts to characterize and treat depression in autism are vital. Emerging work has identified several risk factors in autistic samples including female and non-binary gender identities (6, 7) and increased prevalence over development (8), which mirror findings in the general population. Elevated autistic traits predict depression in this cohort (9) and highlight the importance of investigations into autism-specific factors as potential explanations to increased prevalence rates. One such area of emerging research has focused on repetitive cognitions and behaviors common in autism.

Repetitive behaviors constitute key criteria of autism spectrum disorder [ASD; (10)], and are heterogeneous in nature. Common externalized repetitive behaviors include motor stereotypies (e.g., finger flicking or hand flapping), sensory-seeking, and/or self-injurious behaviors. Repetitive *cognition* often takes the form of narrowed focus on rewarding interests within autistic individuals, but may also include rumination on negative events or perceptions [e.g., “I don't fit in”; (11–14)]. On the basis of factor analyses, restricted and repetitive behaviors and interests in autism are commonly conceptualized as two discrete constructs: Repetitive Sensorimotor (RSM) behaviors and Insistence on Sameness [IS; (15)]. The RSM factor is comprised of motor mannerisms, sensory seeking behaviors, and repetitive use of objects, while the IS factor is comprised of ritualized behavior and distress with changes in routine or expectations (16). While RSM behaviors generally decrease over development within autism (17), IS tends to both persist through later ages and better differentiate autistic individuals from others with developmental disabilities (18). Thus, IS has been described as a “higher-order” behavior that may be more specific to autism (19). However, in other populations in which social communication difficulties and IS occur, they have been positively associated with suicidality (20), thus their role in the pathway to depression in autism may be particularly valuable to investigate.

Recent studies implicate repetitive negative thinking (i.e., rumination) in the onset and maintenance of depressive symptoms in autism (21–23), yet work on IS and depression is limited to two studies (21, 24). Higher IS was positively correlated with depressive symptoms in 28 autistic adults, suggesting that patterns of autism-specific repetitive behavior may be associated with elevated depressive symptoms (24). A second study reported that domains of the autism phenotype (i.e., social communication difficulties, IS), cognitive control, and rumination were robust transdiagnostic predictors of suicide risk in a large and clinically-diverse sample that included autistic adults (25). This background, while minimal, indicates that IS may be positively associated with depressive symptoms in autistic adults and therefore, a worthwhile construct to investigate further. However, findings are limited by smaller samples and/or a lack of empirical tests between RSM behaviors, IS, and depressive symptoms.

One of the reasons that IS might be considered in the causal pathway to depression is that it tends to be marked by negative emotional reactivity in the face of change. Based on research in the general population, neuroticism is a personality factor that is largely described by negative emotional reactivity and plays a significant role in predisposing individuals to depression (26, 27). Of the personality factors, Schriber et al. (28) found that neuroticism may be a more robust discriminant of group membership (autistic vs. non-autistic) that is strongly associated internalizing and externalizing symptoms in autistic samples. Elevated neuroticism may set certain cognitive (e.g., rumination), affective (e.g., emotion dysregulation), and behavioral (e.g., camouflaging) processes in motion, which can contribute to depression (29–31). Indeed, a higher prevalence of neurotic traits in autistic samples (32) may predispose this population to maladaptive cognitive and affective processes that could explain, in part, elevated rates of depression in the autistic community. Neuroticism was found to moderate associations between appraised stress and increased negative affect in autistic adults, particularly in new social situations with unfamiliar people (33). Given that IS constitutes a type of rigid cognitive processing often paired with (or at least measured by) negative emotional reactivity, it may be that neuroticism accounts for a significant portion of an observed relationship between IS and depression in autism. To our knowledge, there are no prior investigations of the role of neuroticism in the pathway from IS to depression in autistic samples.

Following this discussion of IS in the pathway to depression in autism, it is important to note that the role of RSM behaviors and associations with depression in autism has been minimally investigated. To understand the full scope of associations between Criterion B symptoms (i.e., restricted and repetitive behaviors and interests) and depression, an investigation of RSM behaviors is warranted. To date, three studies reported positive associations between repetitive behaviors on the Social Responsiveness Scale, Second Edition [SRS-2; (34)] and depressive symptoms, loneliness, or suicidality in autistic adult samples (35–37). These findings may point to the role of repetitive behaviors in the pathway to depression in autistic adults and support a closer examination of RSM behaviors. In these studies, the SRS-2 provided a summary score of Criterion B symptoms, which does not differentiate repetitive cognitions from behaviors. Therefore, an investigation of IS and RSM behaviors as distinct constructs with potentially different patterns of association with depression in autistic adults will afford more insight of these processes.

Our research team is particularly interested in the role of repetitive negative thinking in the maintenance of depression in autistic individuals; however, we seek here to broaden this line of investigation by examining the relationships between more prototypical repetitive behavior subcomponents (IS and RSM) and depressive symptoms in autistic adults. In the present study, we aim to examine the effects of IS and RSM behaviors on depressive symptoms in autistic adults, and whether observed effects are mediated by additional factors (e.g., neuroticism). We hypothesize that: (1) greater IS scores will be associated with greater depressive symptoms on the BDI-II, but that (2) neuroticism will explain the majority of shared variance across IS and depressive symptoms. An exploratory aim of the present study was to examine the relationship between RSM behaviors and depressive symptoms and determine the degree to which this association was confounded by neuroticism. A second exploratory aim was to examine the degree to which the relationship between neuroticism and depression in this population was moderated by either IS or RSM behaviors. Collectively, these findings answer the overarching clinical question of whether IS and/or RSM behaviors should be further explored in the pathway to depression in autistic adults.

## Materials and Methods

The present study was a secondary data analysis of responses to self-report questionnaires at the same timepoint from autistic adults drawn from the Simons Foundation Powering Autism Research for Knowledge [SPARK; (38)]. This registry is a U.S.-based online community that enables autistic individuals and their families to participate in autism research.

### Participants

Independent autistic adults between 18–45:11 years old were invited to participate in this study *via* the SPARK research portal during winter and spring 2019 as part of a larger study on repetitive thinking and psychopathology in autism (Project Number RM0030Gotham). All individuals self-reported a prior professional diagnosis of ASD or equivalent condition (e.g., Asperger syndrome, PDD-NOS). Though diagnoses were not independently validated, all autistic SPARK participants self-report a professional diagnosis, and the majority of these individuals are recruited from university autism clinics (38, 39). Furthermore, a validation study of self-reported autism diagnoses in individuals recruited from one SPARK clinical site (39) found that 99% of self-reported autism diagnoses could be independently confirmed using data present in participants' medical records. Participants in the current study completed a variety of self-report questionnaires online, including demographic information (e.g., age, gender, educational level) and other clinical variables of interest. Upon completion of the study, participants were compensated with $50 Amazon gift cards. A total of 1,012 individuals enrolled in the larger study, 762 of whom were included in the final cohort of the present study. Participants were excluded from the present study if they: (1) did not self-report a professional diagnosis of ASD, (2) did not complete the self-report questionnaires examined in the current analyses, (3) failed embedded attention checks, or (4) answered “Yes” or “Suspected” to a question regarding a diagnosis of Alzheimer's disease (an extremely unlikely outcome in this 18–45 year old age group). All participants provided informed consent and study procedures were approved by the institutional review board at Vanderbilt University Medical Center. See Table 1 for sample description.

**Table 1:** Sample characteristics.

	***M* (*SD*) or *N* (%)**	**Range**
Age (years)	30.90 (7.05)	18.58–46.08
Sex		
Male	282 (37.0%)	
Female	480 (63.0%)	
Gender		
Male	271 (35.6%)	
Female	414 (54.3%)	
Non-binary	77 (10.1%)	
Educational attainment		
Some high school or less	25 (3.3%)	
High school diploma or GED	143 (18.8%)	
Vocational/trade certificate	36 (4.7%)	
Some college but no degree	201 (26.4%)	
Associate degree	78 (10.2%)	
Bachelor's degree	177 (23.2%)	
Graduate/professional degree	102 (13.4%)	
Race/Ethnicity		
Non-Hispanic white	606 (79.5%)	
Age of ASD diagnosis (years)	19.64 (11.21)	0.50–44.00
Received IEP for ASD	271 (35.6%)	
Received any services for ASD	407 (53.4%)	
Lifetime psychiatric diagnoses		
Unipolar depression	371 (48.7%)	
Bipolar disorder	164 (21.6%)	
Any anxiety disorder	546 (71.7%)	
Number of non-ASD diagnoses	3.26 (2.14)	0–11
Clinical variables		
BDI-II T-score	50.51 (9.44)	31.47–77.22
RBS-R RSM (mean score)	0.69 (0.53)	0.00–3.00
RBS-R IS (mean score)	0.79 (0.61)	0.00–3.00
IPIP-N10 (neuroticism mean score)	3.61 (0.75)	1.20–5.00

*Values are presented as M (SD) for continuous variables and N (%) for categorical variables. ASD, Autism Spectrum Disorder; IEP, Individualized Education Program; BDI-II, Beck Depression Inventory–II; RBS-R, Repetitive Behavior Scale–Revised; RSM, Repetitive Sensory-Motor factor; IS, Insistence on Sameness factor; IPIP-N10, neuroticism scale derived from international personality item pool*.

### Measures

#### Beck Depression Inventory, Second Edition (BDI-II)

The BDI-II is a self-report measure of depressive symptoms in the past two weeks (40). It is comprised of 21 items with severity ratings 0 to 3 for each item. The BDI-II is a widely-used measure of depression in the general population (41), and recently validated for use with autistic adults (42). Using the item response theory-based score calculator provided by Williams et al. (https://asdmeasures.shinyapps.io/bdi_score/), we calculated autism-specific BDI-II latent trait scores, which estimate an individual's standing on the “general depression” factor of the BDI-II. These scores were converted into T-scores with a mean of 50 and standard deviation of 10. Average reliability for these latent trait scores in the current sample was excellent (${\bar{r}}_{xx}=$ 0.90).

#### Repetitive Behavior Scale-Revised (RBS-R)

The RBS-R is a questionnaire typically completed by parents to measure a child's restricted, repetitive behaviors and strong fixed interests commonly associated with ASD (43). For the purposes of the present study, a modified self-report version (44) was administered to autistic adults. The RBS-R contains 43 items organized into six theoretically distinct subscales, although factor-analytic studies typically support a slightly different five-factor solution [e.g., (15, 45)]. Each item is rated on an ordinal scale in which a score of zero indicates that the behavior does not occur and scores of 1–3 indicate the degree of severity (1 = mild, 2 = moderate, 3 = severe) of a behavior, if present. Additionally, the present study utilized the IS and RSM factors outlined by Bishop et al. (15) and further validated for the self-report RBS-R by McDermott et al. (44). The RSM score was comprised of the six items of the stereotyped behaviors subscale and item 43 of the restricted behaviors subscale, whereas the IS score was comprised of two items (items 26–27) from the ritualistic subscale and nine items (items 30–39) from the sameness subscale. The two-factor structure of these items was assessed in the current sample using confirmatory factor analysis [full-information maximum likelihood estimation using the mirt R package; (46)]. This two-factor model fit the current data very well {C_2(14)_ = 21.01, *p* = 0.101, CFI_C2_ = 0.997, RMSEA_C2_ = 0.026, CI_90%_ [0.000, 0.047], SRMR = 0.025}, and model-based reliability (47) was high for both the RSM (ω = 0.860) and IS (ω = 0.941) composite scores.

#### International Personality Item Pool 10-Item Neuroticism Scale (IPIP-N10)

Ten items from the international personality item pool (48) were utilized to form a measure of neuroticism for use in this study (referred to hereafter as the IPIP-N10). These items used stems from the Multidimensional Personality Questionnaire's “Stress Reaction” subscale (49), but unlike the parent scale, they were rated on a five-point Likert scale from *Strongly Disagree* to *Strongly Agree*. Half of the items were reverse-coded, and the mean item score (range 1–5) was used as an overall measure of neuroticism in the current analyses. In the current SPARK sample, model-based coefficients of reliability and general factor saturation were found to be acceptable (ω_T_ = 0.891, ω_H_ = 0.838) when derived from a bifactor model with a common method factor for the reverse-coded items (42).

### Statistical Analyses

Data analyses were performed in the R statistical computing environment version 4.0 (50). Participants with one or two missing responses on self-report measures other than the BDI-II were included by imputing missing item responses with the mean for the scale or subscale to which the item belonged. Missing item responses on the BDI-II (present 2.2% of participants) were accommodated using the full-information item scoring algorithm implemented on the online calculator.

#### Predictors of Depressive Symptoms

To test the first hypothesis of identifying predictors of depressive symptoms on the BDI-II, Bayesian linear regression was conducted with BDI-II scores as the dependent variable, and age, gender (male [baseline] vs. female vs. non-binary/other), educational level [estimated as a monotonic ordinal predictor; (51)], RSM scores, and IS scores as predictors. Univariate associations between BDI-II scores and each predictor were also examined using Bayesian correlations or one-way ANOVAs, as appropriate.

We tested each regression or correlation coefficient against the interval null hypothesis that the true value of β/r lies within the interval [−0.1, 0.1], termed the Region of Practical Equivalence [to zero] [ROPE; (52)]. This ROPE region contains all values that we determined a priori to be too small to be practically meaningful, even if the population slope is non-zero. The bounds of the ROPE were chosen such that this region contained all effects smaller than Cohen's **(author?)** (53) definition of a “small” effect, which we deemed negligibly small. Evidence for or against the interval null hypothesis was quantified using the ROPE Bayes factor [BF_ROPE_; (54)]. Based on guidelines for interpreting Bayes factors (55), BF_ROPE_ values > 3 provide substantial evidence in favor of the alternative hypothesis (i.e., the parameter value is large enough to be practically significant), whereas BF_ROPE_ values < 1/3 provide substantial evidence in favor of the interval null hypothesis (i.e., the parameter of interest is so small that it is practically equivalent to zero). Values between 1/3 and 3 are considered inconclusive. Notably, we also examined whether the 95% highest-density credible interval (CrI) of each regression slope overlapped zero, as this allows for a test of the point null hypothesis typically tested in frequentist linear regression analysis. Additional details about the estimation of these regression models, including choice of prior distributions, can be found below in the Bayesian Model Estimation section.

#### Confounding Role of Neuroticism

To test the second hypothesis of the confounding role of neuroticism in the pathway between IS and depressive symptoms, a second regression was conducted that included all predictors from the baseline model, as well as neuroticism (IPIP-N10) scores. In addition, due to the relatively high correlation between IS and RSM scores in the current sample (r = 0.634, CrI_95%_ [0.586, 0.680]), we performed a sensitivity analysis wherein RSM scores were excluded from the full model, allowing us to examine whether collinearity between RSM and IS was driving a lack of association between IS and depression. All regression parameters in these models were tested against the interval null hypothesis of β = [−0.1, 0.1], with BF_ROPE_ values used to quantify evidence for vs. against this hypothesis.

As an additional exploratory analysis, we also tested whether the relationship between neuroticism and depression was significantly moderated by either IS or RSM scores. To evaluate the evidence both for and against each moderation hypothesis, additional Bayesian regression models were constructed, containing all predictors in the full regression model as well as an interaction term (either RSM^*^neuroticism or IS^*^neuroticism). Models with and without interaction terms were compared using marginal likelihood-Bayes factors (BF-_10_) calculated using Bridge Sampling (56, 57). Similar to BF_ROPE_, BF_10_ values > 3 support the alternative hypothesis (moderation by IS or RSM), values < 1/3 support the null hypothesis (no moderation), and values between 1/3 and 3 provide inconclusive support for or against the moderation hypothesis. In cases where the null hypothesis of no moderation was supported, regression coefficients from the adjusted model were not examined further.

#### Bayesian Model Estimation

In all linear models (including both one-way ANOVAs and regression models), a Normal (0, 0.5) prior was placed on all standardized regression coefficients, with continuous predictors divided by two standard deviations in order to place them on the same scale as coefficients for binary/ordinal predictors (58). This prior was chosen as it assumes *a priori* that 95% of standardized regression slopes fall between −1 and 1. The remaining priors in the model included Student-*t*_3_(0, 2.5) for the regression intercept, half-*t*_3_(0, 2.5) for the residual standard deviation (σ), and Dirichlet (1) (i.e., equal category proportions) for the ordinal education predictor. For Bayesian correlations (based on a multivariate *t* distribution), priors included default Student-*t*_3_ priors on distributional parameters μ and log(σ), a Gamma (2, 0.1) prior on ν (degrees of freedom for the *t*-distribution), and a Lewandowski-Kurowicka-Joe (LKJ) prior on the residual correlation coefficient (59), with parameter η = 2 to reduce the prior probability of extreme correlations. Model parameters were estimated via Markov chain Monte Carlo (MCMC) using the No U-turn Sampler (60), with posterior distributions of each parameter estimated using 24,000 post-warmup MCMC draws from 12 Markov chains. Predictor-level missing data were handled using five-fold multiple imputation, as implemented by the *mice* R package (61). Convergence for each model was confirmed values of Vehtari's $\hat{R}$ convergence diagnostic < 1.01 (62).

## Results

### Predictors of Depressive Symptoms

Bivariate correlations indicated that BDI-II scores were moderately correlated with RBS-R scores on both the RSM (*r* = 0.308, CrI_95%_ [0.241, 0.377], *BF*_ROPE_ = 4.64 × 10^5^) and IS (*r* = 0.376, CrI_95%_ [0.305, 0.438], *BF*_ROPE_ = 1.18 × 10^8^) dimensions. Unsurprisingly, BDI-II scores were also strongly correlated with trait neuroticism (*r* = 0.655, CrI_95%_ [0.612, 0.696], *BF*_ROPE_ > 10^20^). The correlation between BDI-II scores and age was practically equivalent to zero (*r* = 0.070, CrI_95%_ [−0.003, 0.140], *BF*_ROPE_ = 0.045), and while there was a non-zero polyserial correlation between BDI-II scores and level of education (*r* = −0.127, CrI_95%_ [−0.197, −0.053]), the Bayes factor also indicated that this value was likely too small to be of practical significance (*BF*_ROPE_ = 0.281). A Bayesian ANOVA also indicated that BDI-II scores were also significantly higher in both female (β = 0.304, CrI_95%_ [0.155, 0.452], *BF*_ROPE_ = 50.39) and non-binary participants (β = 0.606, CrI_95%_ [0.366, 0.849], *BF*_ROPE_ = 6.89 × 10^3^) compared to males.

To explore whether RSM behaviors and IS predict BDI-II scores after controlling for demographic covariates, a multiple regression with age, education, gender, RSM, and IS was run (see Table 2, Model 1). As expected, IS scores demonstrated a moderate positive relationship with depressive symptoms, greatly exceeding the threshold for practical significance (β = 0.242, CrI_95%_ [0.160, 0.327], *BF*_ROPE_ = 390.6). Alternatively, while there did exist a positive non-zero association between RSM scores and depression, the ROPE Bayes factor indicated that this relationship was small enough to be practically insignificant (β = 0.112, CrI_95%_ [0.031, 0.198], *BF*_ROPE_ = 0.298). Older age, lower educational attainment, female gender, and non-binary gender also demonstrated significantly non-zero associations with depressive symptoms. However, only the effects of female and non-binary gender rose to the level of practical significance (*BF*_ROPE_ > 3), with all other demographic predictors producing regression coefficients that were practically equivalent to zero (Table 2).

**Table 2:** Regression model coefficients and associated Bayesian indices.

	**β_Std_ [95% CrI]**	***P*_d_**	***P*_ROPE_**	***P*_PSig_**	***BF*_ROPE_**
**Model 1: predictive model excluding neuroticism**					
Intercept	0.051 [−0.139, 0.281]	—	—	—	—
Age	**0.088 [0.019, 0.155]**	0.994	0.636	0.364	0.107
Female gender	**0.229 [0.092, 0.371]**	0.999	0.035	0.965	5.29
Non-binary gender	**0.406 [0.175, 0.639]**	>0.999	0.005	0.995	38.87
Education	**−0.064 [−0.110**, **−0.025]**	>0.999	0.938	0.062	0.012
RSM	**0.112 [0.031, 0.198]**	0.996	0.386	0.614	0.298
IS	**0.242 [0.160, 0.327]**	>0.999	<0.001	>0.999	390.6
**Model 2: full model with neuroticism**					
Intercept	0.068 [−0.080, 0.240]	—	—	—	—
Age	0.053 [−0.001, 0.109]	0.970	0.951	0.049	0.010
Female gender	0.095 [−0.022, 0.208]	0.948	0.533	0.467	0.165
Non-binary gender	**0.234 [0.043, 0.424]**	0.992	0.083	0.916	2.07
Education	–**0.043 [−0.078**, **−0.012]**	0.997	0.997	0.003	<0.001
RSM	**0.097 [0.028, 0.164]**	0.997	0.538	0.462	0.161
IS	0.033 [−0.036, 0.107]	0.821	0.966	0.034	0.007
Neuroticism	**0.590 [0.533, 0.650]**	>0.999	<0.001	>0.999	>10^10^
**Model 3: sensitivity analysis excluding RSM**					
Intercept	0.052 [−0.091, 0.217]	—	—	—	—
Age	0.052 [−0.004, 0.108]	0.966	0.953	0.047	0.009
Female gender	0.104 [−0.010, 0.222]	0.961	0.472	0.528	0.208
Non-binary gender	**0.264 [0.071, 0.451]**	0.997	0.046	0.954	3.96
Education	**−0.041 [−0.076**, **−0.010]**	0.996	0.998	0.002	<0.001
IS	**0.089 [0.029, 0.147]**	0.998	0.644	0.356	0.104
Neuroticism	**0.591 [0.533, 0.651]**	>0.999	<0.001	>0.999	>10^10^

*Beta values are presented as posterior medians and 95% highest density credible intervals (CrI). Coefficients with credible intervals that do not overlap zero are bolded. All coefficients were tested against a region of practical equivalence (ROPE) of [−0.1, 0.1]. P_d_, Probability of Direction (the posterior probability of a coefficient being in the same direction as the point estimate); P_ROPE_, Posterior probability of the coefficient falling within the ROPE; P_Psig_, Probability of practically significant effect (the posterior probability that the coefficient exceeds the ROPE in the same direction as the point estimate); BF_ROPE_, ROPE Bayes factor*.

### Confounding Role of Neuroticism

To explore whether the association between IS and BDI-II scores remained practically significant after controlling for neuroticism, the previously described multiple regression model was run with neuroticism added as an additional predictor (see Table 2, Model 2). In this model, higher levels of neuroticism strongly predicted elevated depression symptoms, with this relationship greatly exceeding the threshold for practical significance (β = 0.590, CrI_95%_ [0.533, 0.650], *BF*_ROPE_ > 10^10^). With the addition of neuroticism in the model, IS scores no longer demonstrated a significant relationship with depressive symptoms, and the ROPE Bayes factor demonstrated substantial evidence *against* a practically meaningful effect (β = 0.033, CrI_95%_ [−0.036, 0.107], *BF*_ROPE_ = 0.007). Similar to results from the previous model, RSM scores exhibited a positive nonzero association with depression scores, but the ROPE Bayes factor indicated that this relationship was still small enough to be practically insignificant (β = 0.097, CrI_95%_ [0.028, 0.164], *BF*_ROPE_ = 0.161). Predictors that were found to be too small for practical significance in the first model remained as such in the second model with neuroticism added, including lower educational attainment and age (all *BF*_ROPE_ < 0.010). Additionally, while the effect of female gender was found to be practically equivalent to zero in this model (*BF*_ROPE_ = 0.165), the effect of non-binary gender was still non-zero, although the Bayes factor provided only weak and inconclusive evidence in favor of its practical significance (*BF*_ROPE_ = 2.07). Exploratory moderation analyses found significant evidence *against* interactions of neuroticism with both RSM (*BF*_10_ = 0.006) and IS (*BF*_10_ = 0.005), suggesting that neither RRB construct moderated the neuroticism-depression association.

As RSM and IS scores were highly correlated in the current sample (*r* = 0.634, CrI_95%_ [0.586, 0.680]), a *post-hoc* sensitivity analysis was run in which the full regression model (excluding interaction terms) was re-run without RSM as a predictor (see Table 2, Model 3). This model was investigated in order to confirm that the lack of association between IS and depression after controlling for neuroticism was not due to the large amount of shared variance accounted for by RSM scores. In this model, IS scores did demonstrate a non-zero association with depressive symptoms (β = 0.089, CrI_95%_ [0.029, 0.147], *BF*_ROPE_ = 0.104), but notably, this relationship remained small enough to be practically insignificant. In addition, neuroticism remained a strong and practically significant predictor of depressive symptoms, with the magnitude of this relationship essentially unchanged (β = 0.592, CrI_95%_ [0.533, 0.651], *BF*_ROPE_ > 10^10^). The relationships between depressive symptoms and all demographic predictors were similar in magnitude to those in the previous model (Table 2). Notably, the effect of non-binary gender was slightly larger in this model than in model 2 (Δβ = 0.030), with this coefficient once again exceeding the threshold for practical significance (*BF*_ROPE_ = 3.96).

## Discussion

We sought to determine the association between depressive symptoms and repetitive behaviors characteristic of autism (i.e., insistence on sameness, IS; repetitive sensorimotor behaviors, RSM) in autistic adults, and to investigate whether observed relationships remained significant after controlling for trait neuroticism. Findings from this large sample of autistic adults confirmed both primary hypotheses, as IS exhibited a moderate positive relationship with depressive symptoms but only in regression models that did not account for trait neuroticism. A small positive association between RSM behaviors and depressive symptoms was found in regression models both with and without neuroticism, though the magnitude of this effect was small enough to be practically equivalent to zero. Exploratory moderation analyses did not reveal interactions of neuroticism with RSM nor IS, suggesting that neither RRB construct moderated the observed neuroticism-depression association.

To this point, only preliminary findings from small samples (24, 25) were available to suggest that IS may be related to depressive symptoms in autistic adults. The main contribution of the current paper is the novel finding that, when neuroticism was added to the regression model, the relationship between IS and depressive symptoms was completely eliminated in a large autistic sample. Even when RSM behaviors were removed from the model due to its shared variance with IS, the relationship between IS and depressive symptoms was attenuated to the point that it was not practically significant after accounting for neuroticism. This suggests that more prototypical repetitive cognitions (IS) and/or behaviors (RSM) associated with autism may not constitute independent risk factors to depression in this population.

Elevated neuroticism has been found to underlie internalizing conditions (i.e., depression, anxiety) in neurotypical adults (63) and this study extends that work to autistic adults. Previous work on personality profiles in autism indicates that neuroticism may be more prevalent in autistic adults (32) than the general population, and the current study adds to this early work by uncovering a strong positive association between neuroticism and depressive symptoms specifically, one that eclipses the effect of IS in the pathway to depression. In fact, a closer examination of the IS items on the RBS-R reveals signifiers of negative emotional reactivity (e.g., “disturbed by”, “upset if,” “object to,” or, “difficulty with”), which may explain why these scores do not independently predict depressive symptoms above and beyond levels of trait neuroticism. Simply put, the current findings lead us to believe that it is a predisposition to negative emotional reactivity, rather than the more autism-specific desire for sameness itself, that drives the observed relationship between IS and depressive symptoms.

Although the association between IS and depressive symptoms was explained by neuroticism, the overlap in these constructs may still prove useful for screening and intervention efforts. Though depression measures validated in autistic adults (e.g., BDI-II) are a superior method of depression screening in this population, individuals may receive limited phenotyping data in time-limited clinical and research settings. Therefore, measures of autism features (e.g., SRS-2) that are more commonly employed in routine autism phenotyping may be used to assess IS, which may flag individuals with higher neuroticism to indicate current or future risk for depression. Even without a dedicated depression measure, researchers and clinicians could nevertheless estimate depression risk during their current evaluations, improving mental health service provision without any additional burden on the patient/family members. To guide treatment efforts, IS may also be an important target for treatment planning and coping skills work, as it may represent a frequent obstacle for many autistic adults in regulating emotions and sustaining a healthy mood. In particular, a focus on distress tolerance skills in treatment [e.g., (64)] may be beneficial for autistic individuals with highly reactive IS to learn adaptive responses to the often unpredictable and unstable events in life.

The RSM behaviors on the RBS-R did not demonstrate a practically meaningful association with depressive symptoms. It appears that repetitive *cognitive* processes (e.g., anticipating predictable routines, distress at unstable patterns), rather than repetitive *behavioral* processes (e.g., stereotyped movements, use of objects in a particular way), may be more related to depression in autistic adults. This finding aligns with the work of various research groups on cognitive repetitive processes (e.g., rumination, negative appraisals) in depression in the context of autism (8, 12, 13, 65). Although certain behavioral mechanisms (e.g., decreased physical activity, isolation) have been linked to depression (66, 67), our findings suggest that stereotyped, repetitive behaviors specific to autism may not convey risk for depression in this population. Given limited resources, we might maximize screening and intervention efforts for depression in autism by focusing on repetitive cognition and emotional reactivity rather than manifest repetitive behaviors.

Female and non-binary gender identities were positively and meaningfully associated with depressive symptoms in the baseline regression model without neuroticism, while age and educational level demonstrated associations too small to be practically significant. Not surprisingly, these gender findings mirror those of previous studies in autistic (6, 7) and neurotypical (68) samples. Interestingly, once controlling for trait neuroticism, both associations were attenuated, and only non-binary gender identity independently predicted depressive symptoms. This may suggest that gender differences in neuroticism account for much of the gender differences in depressive symptoms seen in autistic adults, although additional factors beyond trait neuroticism (e.g., gender-based discrimination, systematic prejudice) may further elevate the risk for depression in gender non-binary autistic adults. In fact, gender non-binary autistic adults constituted the most vulnerable cohort in this sample with a greater number of depressive symptoms, a finding which aligns with recent work on gender and mental health in autism (69). As female and gender non-binary autistic adults are at greater risk for depression, yet historically under-represented in autism research (70), our findings reiterate the need for continued investigations of risk/resilience factors and interventions for depression in gender-diverse cohorts. Lower educational level also showed a small and practically insignificant relationship with depressive symptoms in this sample, which adds to the few studies of mixed findings on educational level and depression in autism (71, 72). Although it is likely the true effect of education level on depression is non-zero, the magnitude of this effect in the population is small enough that studies with small or moderate sample sizes are unlikely to be powered to detect it. Furthermore, it is likely that other socioeconomic factors (e.g., region/location, household income, employment status) may be salient risk factors for depression in autistic adults, but warrant further investigation.

## Limitations

In this first investigation of IS and RSM behaviors in the pathway to depression in autistic adults with consideration of trait neuroticism, there are several limitations to consider. First, this sample of autistic adults is comprised of disproportionately more females, adults with higher education levels, and adults with later initial diagnoses of autism as compared to typical clinical convenience samples, as noted in our previous studies (42, 73). Second, given the nature of the SPARK dataset, autism diagnoses were not independently validated and IQ scores were not available to include as a covariate in analyses. Third, the cross-sectional nature of the data prevents us from making causal claims regarding associations between IS, RSM behaviors, and depressive symptoms, as well as inferences about changes in these constructs over time. Fourth, the study sample included only autistic adults and thus, findings cannot be extended to non-autistic populations nor truly concluded to be “autism-specific” without a comparison group. These limitations suggest future directions in the study of repetitive mechanisms and depression in autism. Despite these limitations, the present study advances our understanding of repetitive cognitive and behavioral processes and neuroticism in the pathway to depression in autism. By presenting this work, we hope that researchers and clinicians alike will consider repetitive cognitive processes and neuroticism in studying and treating depression in autism.

## Conclusion

With depression on the rise among autistic adults, research into risk and resilience factors to guide screening and intervention efforts is critical. The extent to which autism-prototypical repetitive cognitive processes (e.g., insistence on sameness; IS) and/or behaviors (e.g., repetitive sensorimotor behaviors; RSM) may be risk factors to depression in autism is unclear. The current analyses from a large autistic adult sample revealed a moderate positive relationship between IS and depressive symptoms; however, neuroticism exhibited a strong positive relationship with depressive symptoms that completely attenuated the effect of IS. The RSM behaviors did not meaningfully predict depressive symptoms. After accounting for the effect of trait neuroticism, a gender non-binary identity conveyed independent risk for depression in this sample, yet all other demographic variables were not meaningfully associated with depression. The findings from this study add to the literature on risk factors in the pathway to depression in autism, with implications for screening and intervention efforts: Even if neuroticism is the underlying trait associated with depression (over IS), the *need for flexibility in daily life* may be the context that frequently evokes negative emotional reactivity in autistic individuals, and thus IS may still represent an important context for skill building and remediation to promote mental health in the autistic community.

## Data Availability Statement

The original contributions presented in the study are included in the article/supplementary files, further inquiries can be directed to the corresponding author. Approved researchers can obtain the SPARK population data set described in this study by applying at https://base.sfari.org.

## Ethics Statement

The studies involving human participants were reviewed and approved by Vanderbilt University Medical Center, Institutional Review Board. The patients/participants provided their written informed consent to participate in this study.

## Author Contributions

KG study concept and design and data collection. JS, ZW, KG, JR, and SM draft manuscript preparation. ZW and JR analysis and interpretation of results. All authors reviewed the results and approved the final version of the manuscript.

## Funding

This work was supported by National Institute of Mental Health Grant R01-MH113576 (KG); National Institute of Mental Health Grant K01-MH103500 (KG); National Institute of General Medical Sciences Grant T32-GM007347 (ZW); National Institute Deafness and Other Communication Disorders Grant F30-DC019510 (ZW); and the Nancy Lurie Marks Family Foundation (ZW). No funding body or source of support had a role in the study design, data collection, analysis or interpretation, decision to publish, or preparation of this manuscript.

## Author Disclaimer

Content is solely the responsibility of the authors and does not necessarily represent the official views of the National Institutes of Health (NIH).

## Conflict of Interest

ZW has served as a consultant for Roche. The remaining authors declare that the research was conducted in the absence of any commercial or financial relationships that could be construed as a potential conflict of interest.

## Publisher's Note

All claims expressed in this article are solely those of the authors and do not necessarily represent those of their affiliated organizations, or those of the publisher, the editors and the reviewers. Any product that may be evaluated in this article, or claim that may be made by its manufacturer, is not guaranteed or endorsed by the publisher.
